# The International Medical Congress

**Published:** 1867-11

**Authors:** Edmund Andrews


### The International Medical Congress.

This body of medical men, selected from the whole civilized world—from San Francisco to Constantinople, at least—assembled at Paris, August 16, 1867, for the first session. The meetings are held in the Ecole de Medicine, the great school of the Paris Faculty of Medicine. In some respects, the French are behind the age in conducting voluntary conventions. There has been very little pains taken to render the advent and the stay of delegates agreeable and convenient. All that free-hearted zeal which welcomes our American Medical Association to its yearly gathering, and strives, in numberless ways, to make the members enjoy their visit, is wanting here. The Parisians seem to suppose that it is a sufficient blessing to outside barbarians to be permitted to come to the Parisian Paradise, and hear the medical angels expound their discoveries. The *concierge* at the gate did not know where the

Secretary was, when he would be in, nor whether the Congress was to have such an officer, until numerous inquiries awakened a suspicion of the fact in his mind. No directions were posted. as to where credentials were to be presented, or names recorded. The delegates ran about the building in a bewildered state, inquiring of each other where the Secretary was, using first one language, and then another, as if a man who did not know a fact in French or English, might possibly be aware of it in Dutch. Finally, the Secretary was discovered in a side apartment, with no clue to its location visible. He looks at the credentials, but keeps no general register or directory in a visible form for reference of delegates. Probably, he has a list somewhere, for the records.

At 2 P. M., the President, Prof. Bouillaud, called the assembly to order. About 700 were present. The President then read a lively and brief address, which showed that he had gathered neither loquacity nor dulness, by his advanced age. He welcomed the delegates to Paris, and pointing to the standards of the various nations, grouped around the walls, he said: "Let us salute these flags, then, by uniting our hands, as these interlaced banners are united, in sign of complete cordiality and entire fraternity." The gracefulness of the figure took the audience by surprise and brought down a storm of applause.

After the close of the address, the reading of the papers commenced. The topic of the day was expressed as follows:

"*Anatomy and Physiology of Tubercle. Tuberculization in the different Countries, and its Influence upon the General Mortality.*" The papers presented, were by Prof. Sangalli, of Pavia; Dr. Villemin, of Paris; Prof. Crocq, of Brussels; Prof. Lebert, of Breslau; Dr. Marmisse, of Bordeaux; Dr. D. Lee, of London; Dr. Larramea, of Bordeaux; Dr. Ullersperger, of Munich; Dr. Homan, of Christiana.

After two papers had been read, it became evident that the session was likely to last many hours, and the audience became restless.

Dr. Van Lohe, a Hollander, rose and began to express his dislike of the order of business and his desire that the Congress should use its rights, and discuss such topics as it deemed best, instead of simply hearing a series of papers read. He commenced as follows:

"Mr. President, is it allowable for me to ask a question?"

The President said: "Certainly, proceed."

He proceeded, therefore, in this wise: "I am a foreigner; I am a Hollander, and, as a Hollander, was sent into this assembly to assist at the Congress. But, up to the present time, I find myself mistaken. It looks to me as if this were not an International Medical Congress, but a court, or a college where the physicians concerned have got together to hear each other read, and for mutual admiration."

The audience waked up at this thunderbolt, and began to applaud the bold Hollander. The President, however, checked him, and courteously reminded him that there was a regular programme of topics, to which it was necessary to conform. Dr. V. L., however, was not to be put down, and proceeded with his speech. The President tried, in vain, to stop him. Finally, the audience deemed that he was going too far, and hissed him down. He took his hat and went out. He re-appeared, however, the next day, and the President offered him an opportunity to finish his remarks, but he did not choose to avail himself of the permission.

August 17, at 8 P. M., the second session commenced, there having been no meeting during the day.

The first paper read was on a new operation for opening abscesses of the liver, which had been practiced in Mexico. The second was by Dr. Galezowski, of Paris, on the alterations of the retina in the tuberculous diathesis. Some fine colored drawings were shown, displaying the increased vascularity of the retina in tubercular meningitis, as seen through the ophthalmoscope.

Another paper was read on the same topic, the two together being illustrated with some forty colored drawings of diseased retinæ. Two papers were also read on the treatment of tubercle. After each reading, time was allowed for discussion, which was embraced with more ease and skill than is usual in the American Medical Association. Each speaker advanced to the tribune, and addressed the Congress in a seated posture, a rather awkward position for a speaker; however, the remarks were brief, vigorous, and lively. There was less stiffness than in American scientific bodies, wit flowed freely, and the Congress laughed with great hilarity.

The discussion on tuberculosis, brought out very contradictory opinions, which are at present irreconcilable.

Mons. Villemin has made elaborate observations and experiments which lead him to believe that tuberculosis is as much a specific disease as syphilis or variola, and that it may be taken by contagion and propagated by inoculation. Mons. Lebert, on the contrary, thinks that there is nothing specific about tubercle, and that its elements are purely inflammatory products. He has made a series of experiments, by which he believes he has proved that tubercle may be produced by injecting into the blood various products of disease, and even mineral substances, such as mercury and carbon. These particles being carried to the lungs by the pulmonary artery, become lodged in the capillaries mechanically, and acting as points of irritation produce tubercles, inflammatory in their real nature, and having all the microscopic and pathological character of the tubercles of consumption.

Between these conflicting doctrines, it seems we must rest, and wait for more light. The general drift of sentiment in the Congress was, that there is no peculiar anatomical or histological element in tubercle, whatever may be true of its diathesis, or of its virus, if any exists.

On the second day, among other topics, came up that of menstruation, as affected by climate, races, etc. Some elaborate statistics were produced upon the subject of the average age at which menstruation commences among different people. M. Langneau gave the following classification of some 15,000 observations:

| Race. | Average age of first menstruation. | | |
|---|---|---|---|
| North Germans, | 16 years, | 9 months, | 16 days. |
| English, | 14 " | 11 " | 2 " |
| French, | 15 " | 1 " | 21 " |
| Southern Asiatics, | 12 " | 11 " | 17 " |

Mr. Robert Cowie, of the Shetland Islands, sent a paper on the "*Prolonged Menstrual Life in the Shetland Islands, and its Relation to Longevity*," in which he shows that the Shetland women retain that function very late in life, the average time of the turn of life with them being from 50 to 54 years. By comparison with Scotland, he shows that this late retention of the menstrual function is also accompanied with an increased longevity. He asserts that the age of commencing menstruation does not differ from that of other parts of the kingdom.

On the third day, Prof. Polli, of Milan, known for his experiments on the antiseptic effects of the sulphites in the inferior animals, read a paper on the use of these articles as medicines in septic diseases, and detailed the Italian experience in their use. Both he and the whole Congress seemed to be in blessed ignorance that this matter had been tested and discussed several years ago in Chicago, and that to Dr. Fisher, Dr. Davis, and to the *Chicago Medical Examiner*, belonged the credit of publicly establishing the practical value of the remedies. If the European savants would regularly take and read some of the American medical journals, they would often find that ideas which are quite new to them have been discussed for years in America.

On the fourth day, was discussed the very important topic of the accidents which follow surgical operations, such as erysipelas, hospital gangrene and pyæmia.

The great murderous sin of European hospitals is want of ventilation. They are afraid of pure air, and, consequently, pyæmia is a fearful surgical scourge. Mr. Gosselin, at the Hopital de la Pitie, innocently reports that he has an epidemic of erysipelas regularly, almost every winter (at the season when the weather causes the windows to be closed,) but seemed to have no suspicion that it might be prevented with ease. He also reports, with the utmost coolness, that of the cases which originate in the hospital every third patient dies. I think this is perfect butchery, and I cannot help comparing it with the better practice in Chicago, where, in Mercy Hospital, deaths from pyæmia and erysipelas have become an almost unknown thing, being completely banished by the simple expedient of opening wide the windows, and keeping every surgical patient, night and day, in a current of pure air. Prof. Paget, of London, has recently experimented in the same way, though he does not seem to be aware that we had settled the matter in Chicago, and published it years ago. His report confirms my experience. He says that the most efficient preventive of pyæmia, is to place the patient in a constant current of pure air, an announcement which ought to strike like a peal of thunder on the ears of European surgeons. The effect of foul air in Paris hospitals is such that they do not dare to close wounds by first intention. I have seen them fill the space between the flaps of an amputation with charpie, as they used to do in the dark ages, and the risk of pyæmia is so great as to render many valuable operations wholly unjustifiable.

The surgeons represented in the Congress generally admitted that ventilation favored escape from pyæmia, but the idea rested in their heads in a very sleepy, foggy manner. Not one of them seemed to be aware of the fact that this cause of death can be completely removed by the means of fresh air. The papers which were read showed that the authors were blindly fumbling about in the materia medica, to find some medicament which would enable them to combat pyæmia without pure respiration. Some used a solution of perchloride of iron, applied to the flaps of amputated cases; others applied solutions of carbolic acid; others employed various measures, all very useful and valuable in themselves; but deadly blunders were committed in this respect, viz: they all invited the profession to rely on these secondary measures, instead of *perfecting the ventilation of the hospitals.*

On the fifth day, came up the famous question, "Is it possible to propose to the various governments efficacious measures for restraining the propagation of venereal diseases?"

This question received all sorts of elucidations except clear ones. Those most interested in the discussion, were evidently of the number of those who desired a system of licensed prostitution. Some of the statistics presented were horribly erroneous, as I happened to know from personal investigations, and, on the whole, the discussion of the real question was loose, wandering, and inconclusive.

After the papers were read, verbal discussion upon the subject commenced. The veteran author on syphilis, Ricord, was in the chair. In a few minutes the hot blood of the disputants got the mastery over parliamentary order, the original question was forgotten, and the speakers plunged headlong into a dispute whether syphilitic inoculation was to be commended or not. M. Ricord, though chairman, took an active part in the contest, and pushed his opponents with a relentless vigor that was refreshing to behold, but not very parliamentary for a presiding officer.

The following gentlemen were named as the committee to address the governments on the subject: Messieurs Hebra, Vienna;

Seitz, Munich; Crocq, Brussels; Seco Baldor, Madrid; Galligo, Florence; Palasciano, Naples; Owre, Christiana; Barbosa, Lisbon; Frerichs, Berlin; Huebbenet, Kiew; Fordyce Barker, New York; Wilson Jewell, Philadelphia; Upham, Boston; R. R. MacIlvaine, Cincinnati; Hingston, Montreal.

The facts brought out before the Congress on the subject of syphilization, may be briefly summed up as follows:

Prof. Boëck, of Sweden, treats secondary syphilis by numerous and long-repeated inoculations of the matter of soft chancre. The plan is this: Several inoculations are made at once upon the patient. When the sores are produced, another set is inoculated, and the first ones destroyed by caustic. Thus crop after crop of chancroids is produced, through a period of several months. At length, after an average of about three hundred and forty-five inoculations, a tolerance is produced, and the patient is found incapable of having any more chancroids. He is then pronounced "syphilized" and cured. During this long treatment, the secondary symptoms generally, or at least frequently, disappear.

Against this treatment, it is urged that some patients have been inoculated to complete insusceptibility to soft chancre, and yet have *failed* to be cured of their secondary symptoms; that thoroughly syphilized women have proved that they were not cured, by bearing syphilitic infants. As to the disappearance of secondary eruptions, it is urged that four months is a long time, and that in such a period these eruptions often recede from view, spontaneously. In a few instances, the most disastrous results, and even loss of life, have followed the treatment.

A banquet was held at the Grand Hotel, by such of the members who chose to subscribe for the purpose. About 200 subscribed. The feast opened with great eclat, and good feeling. M. Bouillaud was in the chair. Several short, pithy addresses were read, an awkward way of delivery in dinner speeches, but still, the time went merrily on. In the midst of the festivities, word was brought that the great surgeon, Velpeau, had just died. This announcement cast a gloom over the assembly, which silenced its mirth and rendered the remainder of the evening a melancholy occasion.

At one of the evening sessions of the Congress, M. Milliot exhibited, upon a cat and a dog, a new method of physical diagnosis of tumors, etc., in the visceral cavities. His plan is to illuminate very powerfully the interior of the stomach, rectum, bladder, or vagina by electric light, and then to observe the translucency of the abdomen, on the same principle that we test the translucency of the scrotal tumor to diagnose hydrocele. For this purpose he introduces into the stomach, rectum, etc., small glass tubes, containing an arrangement of platina wires connected with an electric apparatus. In this way, he produces a powerful interior electric light, which renders the abdomen translucent, like a hydrocele. He hopes to use it for the diagnosis of ovarian cysts, but has not yet brought it to any practical usefulness.

At the close of the Congress, the venerable President pronouuced a brief farewell address, and declared the session finally closed.

[*Chicago Medical Examiner.* EDMUND ANDREWS.

